# No limit in interspecific hybridization in schistosomes: observation from a case report

**DOI:** 10.1051/parasite/2019010

**Published:** 2019-03-01

**Authors:** Jérôme Depaquit, Mohammad Akhoundi, Djamel Haouchine, Stéphane Mantelet, Arezki Izri

**Affiliations:** 1 EA7510 ESCAPE, USC ANSES “VECPAR”, UFR Pharmacie, Université de Reims Champagne-Ardenne France; 2 Laboratoire de Parasitologie-Mycologie, Hôpital Maison Blanche Reims France; 3 Département de Parasitologie-Mycologie, Hôpital Avicenne AP-HP Bobigny France; 4 Unité des Virus Emergents (Université Aix-Marseille– IRD 190 – Inserm 1207 – IHU Méditerranée infection) Marseille France

**Keywords:** *Schistosoma mansoni*, *S. haematobium*, Hematuria, Hybrid, phylogenetic distance

## Abstract

Schistosomiasis is one of the most significant parasitic diseases of humans. The hybridization of closely related *Schistosoma* species has already been documented. However, hybridization between phylogenetically distant species is unusual. In the present study, we characterized the causative agent of schistosomiasis in a 14-year-old patient with hematuria from Côte d’Ivoire, using morphological and molecular approaches. A 24-hour parasitological examination of urine showed the presence of numerous eggs (150 μm long × 62 μm wide) with a lateral spine (25 μm), identified morphologically as *Schistosoma mansoni*. Examination of stools performed on the same day found no parasites. The urine and stool examinations of the patient’s family members performed two weeks later showed neither parasites nor hematuria; but in contrast, many *S. mansoni* eggs were found again in the patient’s urine, but never in his stools. Conventional PCRs were performed, using two primer pairs targeting 28S-rDNA and COI mtDNA. The 28S-rDNA sequence of these eggs, compared with two reference sequences from GenBank demonstrated a hybrid with 25 double peaks, indicating clearly hybrid positions (5.37%) between *S. mansoni* and *S. haematobium.* Similarly, we identified a unique *S. mansoni* COI sequence for the two eggs, with 99.1% homology with the *S. mansoni* reference sequence. Consequently, this case was the result of hybridization between an *S. haematobium* male and an *S. mansoni* female. This should be taken into consideration to explore the elimination of ectopic schistosome eggs in the future.

## Abstract (fr)

La schistosomiase est l’une des maladies parasitaires les plus importantes chez l’Homme. L’hybridation d’espèces de *Schistosoma* très apparentées a déjà été documentée. Cependant, l’hybridation entre espèces phylogénétiquement éloignées est inhabituelle. Dans le cas que nous rapportons, nous avons caractérisé l’agent responsable d’une bilharziose urinaire chez un patient de 14 ans originaire de Côte d’Ivoire à l’aide d’approches morphologiques et moléculaires. Un examen parasitologique effectué sur des urines de 24 heures a révélé la présence de nombreux œufs (150 μm de long × 62 μm de large) à éperon latéral (25 μm), identifiés morphologiquement comme appartenant à l’espèce *Schistosoma mansoni*. L’examen parasitologique des selles effectué le même jour n’a révélé aucun parasite. Les examens d’urines et de selles des membres de sa famille effectués deux semaines plus tard ne montraient aucun parasite ni hématurie, mais en revanche, de nombreux œufs de *S. mansoni* ont été retrouvés dans l’urine du patient, mais jamais dans ses selles. Les PCR conventionnelles ont été réalisées à l’aide de paires d’amorces ciblant l’ADNr 28S et l’ADNmt (COI). L’analyse de la séquence de l’ADNr 28S de ces œufs, comparée à deux séquences de référence de GenBank a montré un hybride avec 25 doubles pics indiquant des positions clairement hybrides (5,37 %) entre *S. mansoni* et *S. haematobium*. De même, nous avons identifié une séquence COI unique pour les deux œufs, présentant une homologie de 99,1 % avec la séquence de référence de *S. mansoni*. Par conséquent, ce cas est le résultat d’une hybridation entre un mâle *S. haematobium* et une femelle *S. mansoni*. Ce phénomène devrait être pris en compte pour explorer les éliminations atypiques d’œufs de bilharzies à l’avenir.

## Introduction

Schistosomiases are a group of neglected tropical diseases caused by dioecious trematode parasites of the genus *Schistosoma*. Based on a report of the World Health Organization (WHO), it is one of the most important tropical diseases, infecting more than 250 million people, mainly in sub-Saharan Africa [30].

The hybridization of closely related *Schistosoma* species has already been demonstrated in the laboratory [23], as well as in nature [8]. Nevertheless, hybridization between two major causative agents of intestinal and urinary schistosomiasis in Africa, *Schistosoma mansoni* and *S. haematobium,* has rarely been described [20] and was very recently observed in a patient originated from Côte d’Ivoire [19]. Hybridization between these two species is very surprising due to the high phylogenetic distance between them [15]. However, some cases of mixed infections have been reported in Mali [17], Senegal [24], and Kenya [11]. The elimination of ectopic *S. mansoni* eggs suggested possible hybridization between these two parasitic species, which were documented in humans in Cameroon [8], or in cercaria in Senegal [15].

In the present study, we report the case of a patient from Côte d’Ivoire, excreting living *S. mansoni*-like eggs in his urine without any excretion in his stools. This evidence, together with molecular characterization of some eggs, opens up a discussion on hybridization phenomenon between these genetically distant species. Although this case occurred 10 years ago, we decided to report it considering the invasiveness, frequency and persistence of schistosomiases caused by various hybrids [3, 4, 19, 27].

## Case report

**Figure 1:**
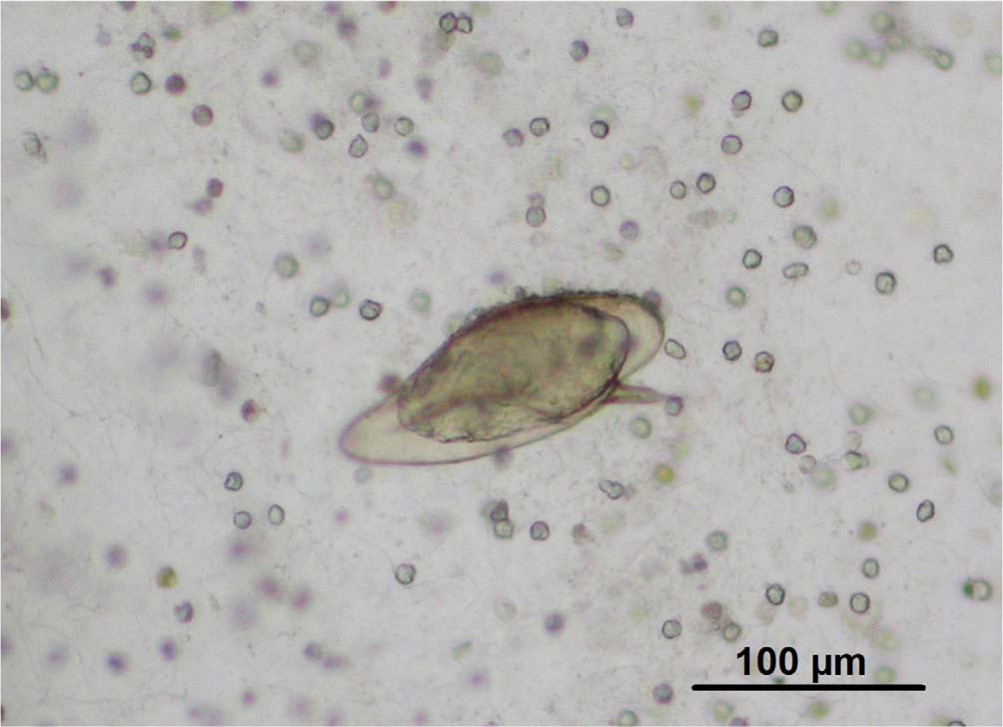
A hybrid *S. mansoni*/*S. haematobium* egg excreted in the urine of the patient.

In March 2008, a 14-year-old schoolboy (weight: 45 kg) who lived with his family in the suburbs of Paris, was referred by his doctor to the Parasitology–Mycology department of the Avicenne Hospital for hematuria and suspected urinary schistosomiasis. His parents are from Côte d’Ivoire, a country where he regularly went during school holidays. The last stay took place in the region of Divo (in south of the country) for 47 days from July 5 to August 20, 2007. The hematuria appeared one week after his return, without other symptoms. The 24-hour parasitological examination of urine performed on March 6, 2008, showed the presence of 35 eggs identified morphologically as *S. mansoni*. A total of 10 eggs were measured and their mean values were 150 μm long (156–146 μm, standard-error 3.20 μm)/62.5 μm wide (60–66 μm, standard-error 1.73 μm), and a 25 μm lateral spin (23.5–27.5 μm, standard-error 1.35 μm). These eggs contained mostly a living miracidium (Fig. 1). All the eggs exhibited the same shape. No terminal spined egg has been observed. Examination of stools performed on the same day found no parasite.

Based on this unusual result, the patient together with six members of his family were called again for urine and stool examinations on May 5, 2008. No parasites were found in the stools and urine of family members. Furthermore, no hematuria was observed among them. In contrast, about 30 *S. mansoni* eggs were found again in the patient’s urine, but not in his stools.

Based on blood cell count, hypereosinophilia (1200 eosinophils/mm^3^) was found in the patient’s blood sample. In addition, no renal or urinary calcium opacity was observed, according to radiography of the abdomen without preparation. Echography examination showed localized bladder nodular thickening of 15 mm. The patient was then treated orally with a single dose of three tablets of praziquantel (1800 mg). The patient was examined again 18 months later. Urine and stool examinations were negative. The presence of living eggs located in the bladder plexus was confirmed by visualization of a bladder nodule on echography, which demonstrated active infestation in the urine.

## Materials and methods

Urine and stool examinations for *Schistosoma* eggs were the primary methods, performed for diagnosis of suspected *Schistosoma* infections. For this purpose, a 24-hour urine sample of the patient was collected, decanted and the first infused drops were subjected to microscopic examination at 100× and 400× magnifications. Stool examination for eggs and parasites was carried out, using the merthiolate-iodine-formaldehyde (MIF) stool concentration technique [1]. Furthermore, two single eggs were isolated, using a micropipette under a stereomicroscope. The eggs’ DNA was extracted, using the Qiamp DNA Mini Kit (Qiagen, Germany). Conventional PCRs were performed, using C2’b and D2 primers for amplification of the D2 domain of 28S rDNA [25], and SchistoCox1-5′ and SchistoCox1-3′ primers for mtDNA COI amplification [35]. The latter is of maternal inheritance, which mainly supports DNA barcoding [13]. Amplicons were analyzed, using electrophoresis on 1.5% agarose gel, containing ethidium bromide. Moreover, amplicons were directly sequenced in both directions, using the same primers used for PCR.

Obtained sequences were edited by Staden package software, and compared with homologous sequences in GenBank, using the Basic Local Alignment Search Tool (BLAST) (www.ncbi.nlm.nih.gov/BLAST). Sequences were aligned with BioEdit v7.0.0 software [12]. Genetic distances and ML trees were obtained, using the HKY85 model on all codon sites, and MEGA7 software [18] was used for those containing gaps.

## Results

Two fragments of 465 and 849 base pairs were amplified separately, using D2 and COI primer pairs, respectively, and visualized, using gel electrophoresis. Then, the sequences of these two egg samples were compared with those deposited in the GenBank.

Regarding the D2 fragment, there were 25 polymorphic position sites between *S. mansoni* (GenBank accession number AY157173) and *S. haematobium* (GenBank accession number AY157263). These reference sequences were selected because they were obtained from strains strongly identified and colonized under laboratory conditions. The D2 sequences from the two eggs showed 25 double peaks of equal intensity in the chromatogram at the same position of the referent sequences, clearly indicating hybrid positions between *S. mansoni* and *S. haematobium*

The D2 sequences included 25 double peaks of equal intensity, clearly indicating hybrid positions (5.37%) between *S. mansoni* and *S. haematobium*.

The sequence of amplified mitochondrial marker (COI) was assessed, in order to explore the ascendance of this hybrid. We compared 849 bp of the COI sequence with those of *S. mansoni* and *S. haematobium* deposited in GenBank (accession numbers FN364013 and AY157209, respectively). We identified for the two eggs a unique *S. mansoni* COI sequence (99.1% homology with the *S. mansoni* referent sequence FN364013). We analysed only 451 bp, in order to compare our sequence to 32 others deposited in GenBank, to calculate pairwise distances (Table 1) and build maximum likelihood trees.

**Table 1:** Estimates of overall evolutionary divergence across sequence pairs including all nucleotides. The values in black are related to distances between *Schistosoma* species based on D2 sequences. The values in red are related to distances between *Schistosoma* species based on COI sequences. The COI intraspecific variability within *S. mansoni* excluding the hybrid processed in the present study is indicated in green.

	*S. mansoni*	*S. haematobium*	*S. bovis*	*S. curassoni*	*S. intercalatum*	*S. leiperi*	*S. margrebowiei*	*S. mattheei*	*S. rodhaini*
*S. mansoni*	0.027	0.180	0.195	0.208	0.190	0.185	0.202	0.180	0.118
*S. haematobium*	0.053		0.114	0.117	0.113	0.098	0.14	0.142	0.152
*S. bovis*	0.056	0.004		0.058	0.120	0.120	0.140	0.131	0.167
*S. curassoni*	0.058	0.006	0.002		0.133	0.113	0.129	0.138	0.173
*S. intercalatum*	0.056	0.004	0.000	0.002		0.098	0.127	0.149	0.169
*S. leiperi*	0.056	0.017	0.013	0.015	0.013		0.138	0.140	0.151
*S. margrebowiei*	0.060	0.026	0.024	0.026	0.024	0.028		0.164	0.176
*S. mattheei*	0.060	0.013	0.009	0.011	0.009	0.013	0.028		0.158
*S. rodhaini*	0.009	0.054	0.052	0.054	0.052	0.052	0.058	0.056	

**Figure 2:**
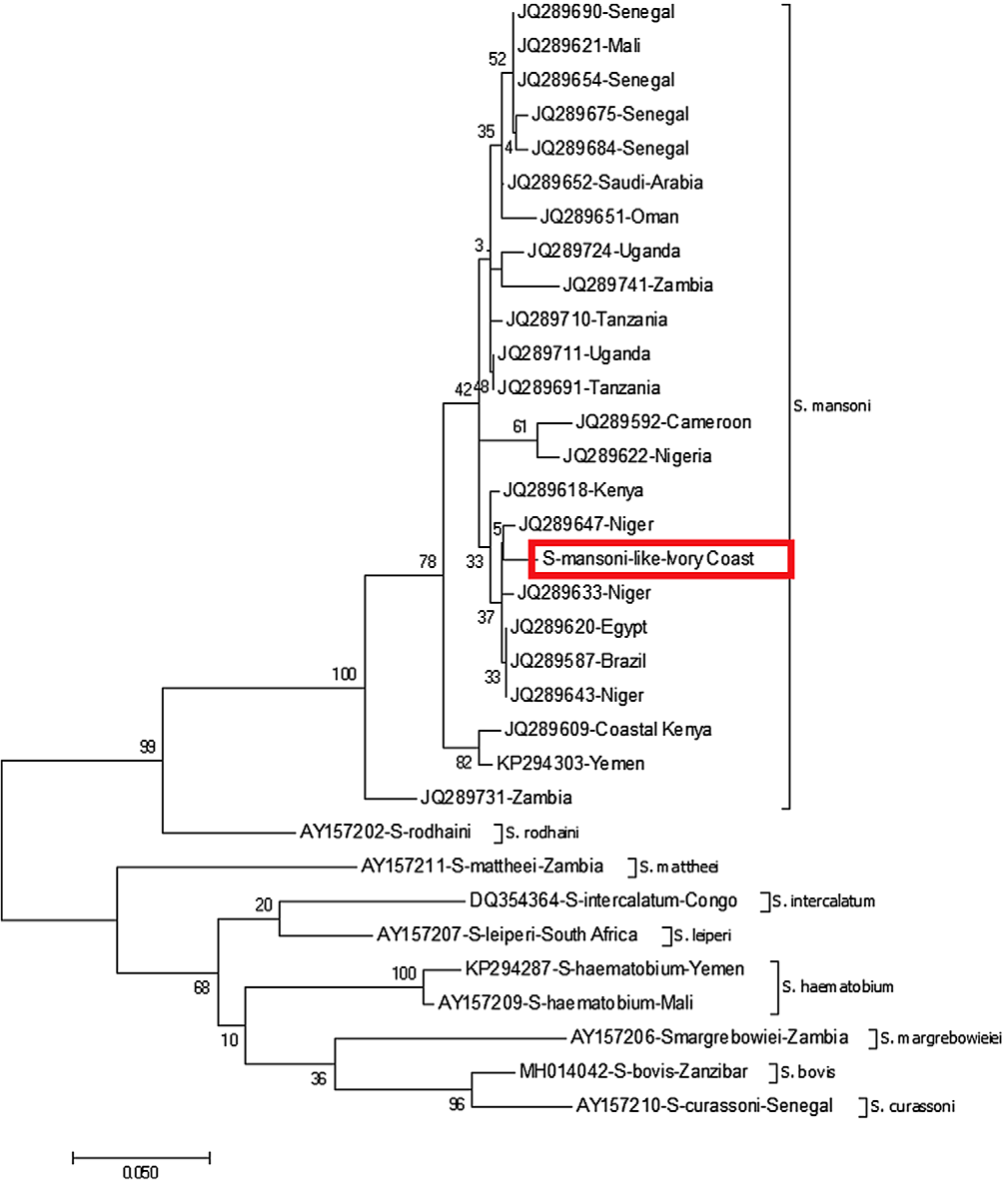
ML tree built on COI mtDNA sequences of *Schistosoma* spp. The percentage of trees in which the associated taxa clustered together is shown next to the branches. The tree is drawn to scale, with branch lengths measured in the number of substitutions per site.

The specimens we processed in the present study based on COI mtDNA sequences, are perfectly included in the *S. mansoni* branch (Fig. 2).

## Discussion

Hybridization of parasites is an emerging public health concern worldwide. This phenomenon can have major impacts on the parasite-host relationship, as well as the epidemiology and course of disease. It can potentially lead to the evolution and emergence of new parasitic microorganisms and consequently new diseases [9, 16]. The exchange of new genes may affect the pathogenicity and resistance of the parasites, and potentially complicates control measures [36]. For instance, *S. haematobium* is unable to develop solely in sheep [33], but its hybridization with *S. mattheei* simplifies the propagation in the mentioned host [32].

Hybridization is a frequent phenomenon between closely related species, such as among “Haematobium group” or between *S. mansoni* and *S. rodhaini*, but hybridization between *S. mansoni* and *S. haematobium* is reported very rarely, and is poorly documented. The eggs with lateral spins were first reported in the human bladder in 1884 by Sonsino [31], and then in 1886 by Belleli [2] who observed a purely *S. mansoni* bladder infection in Egypt. Since the 1900s, several authors have reported the presence of *S. mansoni* eggs in the urine [6, 10]. More recently, the presence of this species was documented frequently in Africa [22, 34]. In 1991, Ratard and colleagues [28] observed patients excreting *S. mansoni* eggs in the urine in the north of Cameroon. A decade later, in agreement with Ratard’s observation, a possible hybridization between *S. haematobium* and *S. mansoni* was strongly suspected in Cameroon [8], and proven in Senegal [15], whereas it has recently been observed in Côte d’Ivoire [19].

Based on comparison of the mitochondrial sequences of the hybrid specimens with those of *S. mansoni* (FN364013), the eggs excreted by the young boy seem to be the consequence of hybridization between a female *S. mansoni* and a male *S. haematobium*. However, it is impossible for us to predict if this child had already become infected by hybrid cercariae or if mating of the male *S. haematobium* and the female *S. mansoni* occurred in his body. A possible route for this hybridization is that the male transported the female to the usual location of *S. haematobium*. However, considering the multiple repeated cases of hybridization between the indicated species in Africa, this case should be taken into consideration in future microscopic and molecular identification of *Schistosoma* parasites.

Hybrids in *Schistosoma* are troublesome. They are well adapted to intermediate hosts in endemic foci of schistosomiasis, like in Senegal, where they seem to be able to modify the epidemiology of the disease [5, 29], maybe due to higher competitiveness. Moreover, they can spread and become invasive populations, like in Corsica, where a recent outbreak was the consequence of hybrids between *S. haematobium* and *S. bovis* [26]. The hybridization between *S. haematobium* and *S. bovis* is not surprising because they are two closely related species. Until recently, the hybridization host of the latter species remained enigmatic since these two species did not have the same final host; adults could not theoretically meet. However, it has recently been shown that small rodents could be their definitive hosts where this hybridization could occur [7]. For *S. haematobium* and *S. mansoni*, their genetic distance is greater, and consequently their hybridization more surprising, due to the significant phylogenetic distance [21, 37]. However, the chance of adults meeting is high because of the sympatry of both *S. haematobium* and *S. mansoni* and the high number of co-infections in some areas. To date, nothing is known about the possible intermediate hosts of such hybrids. *S. haematobium* exclusively infects *Bulinus* spp., whereas, *Biomphalaria* acts as an intermediate host for *S. mansoni*. The spread of these hybrids will be correlated to the distribution of their intermediate hosts. The use of new low-cost and high throughput tools like MALDI-TOF MS [14] to detect such hybrids could be helpful to explore this challenge.
